# Impact of Climate Change on Eye Diseases and Associated Economical Costs

**DOI:** 10.3390/ijerph18137197

**Published:** 2021-07-05

**Authors:** Lucía Echevarría-Lucas, José Mᵃ Senciales-González, María Eloísa Medialdea-Hurtado, Jesús Rodrigo-Comino

**Affiliations:** 1 Ophthalmology Service of Axarquía Hospital, 29700 Vélez-Málaga, Spain; luciaechevarria98@gmail.com (L.E.-L.); medialdeah@gmail.com (M.E.M.-H.); 2 Department of Geography, University of Malaga, 29010 Málaga, Spain; senciales@uma.es; 3 Department of Regional Geographical Analysis and Physical Geography, University of Granada, 18010 Granada, Spain; 4 Department of Physical Geography, University of Trier, 54296 Trier, Germany

**Keywords:** ocular diseases, environmental factors, climate change, economic impact, Southern Spain

## Abstract

Climate change generates negative impacts on human health. However, little is known about specific impacts on eye diseases, especially in arid and semi-arid areas where increases in air temperatures are expected. Therefore, the main goals of this research are: (i) to highlight the association between common eye diseases and environmental factors; and (ii) to analyze, through the available literature, the health expenditure involved in combating these diseases and the savings from mitigating the environmental factors that aggravate them. Mixed methods were used to assess the cross-variables (environmental factors, eye diseases, health costs). Considering Southern Spain as an example, our results showed that areas with similar climatic conditions could increase eye diseases due to a sustained increase in temperatures and torrential rains, among other factors. We highlight that an increase in eye diseases in Southern Spain is conditioned by the effects of climate change by up to 36.5%; the economic burden of the main eye diseases, extrapolated to the rest of the country, would represent an annual burden of 0.7% of Spain’s Gross Domestic Product. In conclusion, the increase in eye diseases has a strong economic and social impact that could be reduced with proper management of the effects of climate change. We propose a new concept: disease sink, defined as any climate change mitigation action which reduces the incidence or morbidity of disease.

## 1. Introduction

The Intergovernmental Panel on Climate Change 2014 Report on Human Health established three basic prospects through which climate change will affect human health [1]: (i) direct impacts, related to changes in the frequency of extreme events, including heatwaves, droughts and heavy rain; (ii) effects on natural systems, such as changes in disease vectors, waterborne diseases and air pollution; and, (iii) strongly conditioned effects by mismanaged human systems, such as labor impacts, malnutrition and mental stress. The third objective of the 2030 UN’s Agenda for Sustainable Development (https://www.un.org/sustainabledevelopment/es/health/ accessed on 13 March 2021) seeks to ensure a healthy life and promote the well-being of all ages, recognizing that further efforts are needed to address and eradicate many health problems.

Concerning direct impacts, Global Warming is a progressive trend with variable intensity all over the world. Thus, heatwaves are strongly associated with mortality increases. Christidis et al. (2012) [2] pointed out that extreme heat events increased by fourfold in Europe between 1999 and 2008, causing in some countries about 15,000 deaths [3]. Several scholars have stated that Global Warming is also responsible for an increase in cardiovascular, respiratory and kidney diseases, besides an increase in forest fires which are associated with an increase in smoke-related morbidity and mortality [4,5]. In terms of eye health, there is evidence of the effect of heat on the inflammatory response of the corneal cells [6]. Furthermore, this has been related to the predisposition to infectious viral processes such as herpes and viral conjunctivitis, besides bacterial and fungal processes, in addition to allergies [7,8]. Moreover, van der Leun et al. (2008) [9] noted that an effective dose of ultraviolet radiation (UVR) could increase by 2% for every degree Celsius as the temperature rises. This phenomenon could also suppress cell immunity and increase sensibility to infections, facilitating the activation of latent viruses [10,11]. Additionally, increasing UVR, in conjunction with summer thermal maximum, is related to the increase of ocular tumors and cataracts [12,13,14,15], along with an increased risk of retinal detachment [16].

Rising temperatures recorded in arid and semi-arid areas such as Southern Spain are common throughout the Mediterranean region [17]. Moreover, some estimations claim that mean air temperatures and solar radiation values may continue rising [18,19], besides facing an increasing frequency of heavy rain and floods [20,21], elements which have well-documented effects on infectious diseases [22].

The alteration of natural systems favors an increase in the incidence of vector-borne diseases and even their appearance in areas where they did not exist before. Some diseases eradicated in Spain such as malaria or trachoma could reappear [23,24]. Some other diseases could appear or increase their currently scarce numbers: dengue and hemorrhagic fever with renal syndrome (increasing with temperatures, rainfall and humidity), tick-borne diseases (increasing with temperatures), and other pests such as Chikungunya, Japanese encephalitis, Rift Valley fever [25,26,27,28]), or West Nile Fever [29,30]. These all have important and well-known effects on eye structures [31,32,33,34,35].

Air quality is also a conditioning factor for human health, especially concerning other pollutants than carbon dioxide. Among these, tropospheric ozone must be highlighted as a common urban pollutant [36] that may be magnified by heatwaves [37,38]. Moreover, acute pollution events, which are related to high atmospheric stability, heatwaves and droughts, have been associated with high levels of aerodynamic particles (PM_10_, PM_2.5_) involving severe outbreaks of premature mortality [4], which also are being studied in relation to the COVID-19 outbreak [39]. Additionally, allergenic particles have increased due to warmer atmospheric conditions, promoting dermatitis, asthma attacks, allergic rhinitis and allergic conjunctivitis [40]. Finally, air quality has been related to several complications of the ocular surface, implying damages to the cornea and the conjunctiva, and several disorders such as cataracts, conjunctivitis, glaucoma, and dry eye, among others [41,42,43].

Furthermore, as an effect of climate change, mismanaged human systems lead to malnutrition, work-related health problems and mental disorders. Thus, malnutrition or undernourishment is a direct effect of changes in harvest because of temperature changes or rainfall regimes, especially in those areas where agriculture and livestock have naturally low productivity [44,45] and are sensitive to price fluctuations. This leads to the consumption of food without crucial active principles of nourishment [46]. Malnutrition is implied in xerophthalmia, a disease caused by a deficiency of Vitamin A, which provokes blindness in children under the age of five, with high mortality [47,48]. Malnutrition is also responsible for ocular problems in adulthood [49], causing diseases such as AMD (Age-related Macular Degeneration), cataracts, or glaucoma [50]. In addition, outdoor workers (farmers, building and clean-up workers) directly suffer heatwaves, reducing their productivity in order to avoid them [51,52]. Moreover, they also suffer vector-borne outbreaks, psycho-physical damage and secondary ocular diseases such as pterygium, glaucoma and retino-choroidal injuries. Farmers have eight times higher possibilities of suffering ocular damage than other professionals. 

However, information on the long-term impact of climate change on eye diseases regarding their economic effects is lacking. Therefore, the main aims of this investigation are (i) to demonstrate the association between common eye diseases and environmental factors, taking as a reference two highly populated cities in Southern Spain (Málaga and Almería), which are considered highly vulnerable areas to climate change; and (ii) to estimate, using the available literature, the health expenditure involved in combating these diseases and the savings that would be made by mitigating the environmental factors that aggravate them. To achieve these goals, mixed methods to assess the cross-variables (environmental factors, eye diseases and health costs) were used. We hypothesize that current research could focus on the work of those interested in the role of climate change regarding ocular health and may be useful to policymakers in order to explore associated economic damages of a policy of non-intervention concerning climate change.

## 2. Methods

Mixed methods were used to highlight the cross-variables for southern Spain: environmental factors, eye diseases and health costs.

Thus, climate data from two cities in Southern Spain (Málaga and Almería) have been taken and analysed for comparison and extension with previous research. Temperature and rainfall datasets to assess the climate trends of Málaga and Almería urban areas were used (574,674 and 198,533 inhabitants, respectively) [53]. Data from, at least, the last 30 years have been used to analyze precipitation, average, minimum and maximum temperatures and humidity. The climate stations selected were Málaga and Almería Airports due to the available long-term data series, as well as the location near to the sea. Both are representative examples of semi-arid (Málaga) and arid (Almería) conditions in southern Spain. The dataset has been extracted and analysed from the REDIAM (Environmental Information Network of the Andalusian Regional Government (http://www.juntadeandalucia.es/medioambiente/site/rediam accessed on 9 January 2021) database. Finally, the results were compared to and contrasted with the IPCC reports to verify whether or not they are consistent with the internationally established outlook for the region [1,54,55,56].

Then data on the most common eye diseases in the region were reviewed and their attribution to environmental variables associated with climate change analyzed.

The most frequent ocular pathologies associated with environmental variables that are undergoing special modifications in arid and semi-arid areas such as the south of Spain [57,58,59] are summarized as follows.

### 2.1. Cornea, Sclera and Conjunctive

Several pathologies of these ocular structures are related to a wide range of environmental variables; they are divisible into three groups:

(A). Increasing inflammation: Allergic Keratoconjunctivitis, Marginal Keratitis, Dry Keratitis, Chronic Episcleritis, Corneal Metaplasia, and Pterygium;
(B). Increasing infections and superinfections: Corneal and Conjunctival Herpes simplex and Herpes Zoster, Viral Keratoconjunctivitis, Accidental Corneal Fungal Injuries, Infectious Corneal Injuries, non-fungal injury by Contact Lens;
(C). Tumor processes in Cornea, Conjunctive and Ocular Annexes: Epidermoid Neoplasia of Ocular Surface, Basal Cell Carcinoma of Eyelid.

### 2.2. Glaucoma

This disease has been related to environmental variables, highlighting temperatures, UVR and pollutants. It is divisible as follows:

(D). Acute Glaucoma;
(E). Chronic open-angle Glaucoma.

### 2.3. Cataracts

This visual impairment is also related to environmental variables, especially solar radiation. It is divisible into two groups:

(F). Early Cortical and Subcapsular Cataracts;
(G). Pseudo-exfoliation syndrome.

### 2.4. Tumor Processes in the Choroid, Iris and Ciliary Body

(H). Uveal Melanoma is the main disease of this group related to environmental factors. Several authors have related it to exposure to UVB radiation [12,60,61].

### 2.5. Uveitis (Intraocular Inflammatory Processes)

Four different uveitic processes associated with various pathologies have been related to a wide range of environmental factors, as can be seen in Table S1. These groups are:

(I). Infectious uveitis: Toxoplasmosis, Tuberculosis, Campylobacter, Chlamydia, Ocular Herpes simplex (VHS) and Zoster (VHZ), West Nile Fever, Borreliosis and Rickettsiosis, Shigellosis, Salmonellosis; the World Health Organization offers information about several pathogens and their infectivity [62,63], including those which are engaged in ocular diseases; a higher frequency of infectious uveitis has been highlighted during the summer [64], plus a change in patterns, incidence and prevalence [65].
(J). Non-infectious uveitis associated with systemic diseases: Rheumatoid arthritis (RA), Ankylosing spondylitis (AS), Sarcoidosis, Multiple Sclerosis (MS), Inflammatory bowel diseases (IBD—Crohn and ulcer colitis), Behçet’s disease, Giant cells arteritis (Horton’s disease), Necrotizing systemic vasculitis.
(K). Connective tissue diseases: Systemic Lupus Erythematosus (SLE), Dermatomyositis.
(L). Uveitis merely as ocular disease, without associated systemic pathologies: Fuchs’ heterochronic Uveitis, Posner-Schlossmann syndrome, Intermedia uveitis and pars planitis, Birdshot choroidopathy, Vogt-Koyanagi-Harada syndrome (VKH), White Dots Syndromes [66].

### 2.6. Retina

Finally, several retinal injuries have been related to environmental factors, with particular emphasis on sunlight and UVR, among others. These have been classified into four groups:

(M). Tractional Retinal Detachment and Retinal Tears.
(N). Posterior Vitreous Detachment.
(O). Age Macular Degeneration (AMD).
(P). Central Serous Choroidopathy.

Finally, information on the costs of these diseases was examined in the available literature, resulting in the attribution of the health costs of eye diseases to climate change. An economic assessment of the estimated costs of the most frequent ocular pathologies has been developed. Sun exposure excess, high temperatures, pouring rain, wind or pollution effects, among others, are approachable variables with regard to land management solutions. By looking at the most common ocular diseases and their high impact on health, it is possible to see the magnitude of the problem.

Thus, where possible, the average cost of treatments for each eye disease in Spain has been analysed. When this was not possible, references were taken from Europe or, failing that, from the United States.

The environmental factors that predispose or exacerbate the different ocular pathologies have been consulted, searching the available literature especially for the percentage of attribution of environmental factors related to climate change to these pathologies. From this, we deduced the increased costs of failing to control environmental factors, producing a final sum of the economic cost of climate change-related eye pathologies.

Likewise, climate change mitigation measures and the economic weight that carrying them out would imply have been analysed in order to assess the final economic balance, comparing the benefits of adaptation with those of mitigation; the former would imply seeking additional resources to better address diseases, reducing vulnerability and increasing resilience capacity; while the latter would imply a reduction in the danger and risk associated with environmental variables [67,68,69].

## 3. Results

### 3.1. Background of Climate Conditions in Almería and Málaga

**Figure 1:**
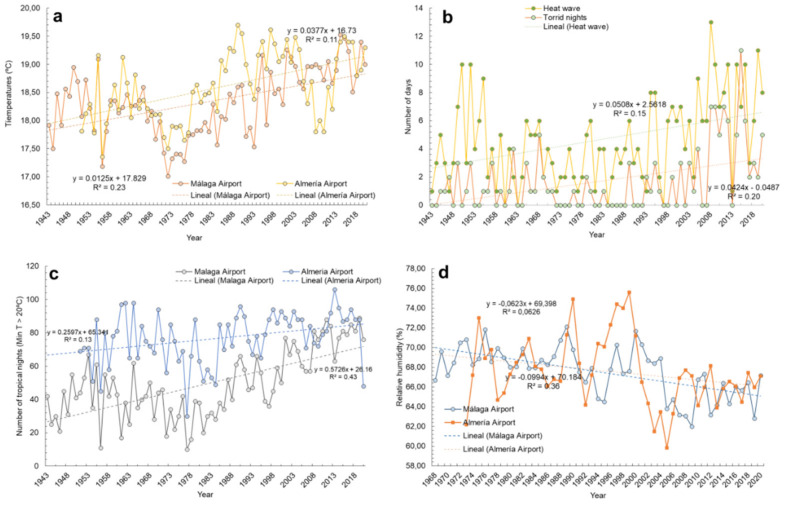
Climate evolution and trends of Almería and Málaga airport meteorological stations. Mean annual air temperatures (**a**), number of heatwaves and torrid days (**b**), number of tropical days (**c**) and average relative humidity (**d**). Own elaboration from REDIAM data.

As in other parts of the Mediterranean Region [70], the observed trends in these two cities can be synthesized as follows (for more information, see Supplementary Material Tables S1 and S2): a sustained increase in air temperatures of about 0.1–0.4 °C/decade (Figure 1a), which has changed the Köppen-Geiger classification for Almería Airport from BSk to BSh (warmer sub-desertic climate), and the average annual temperature for Málaga Airport from 17.96 °C (1961–1990) to 18.76 °C (1991–2020); the higher frequency of heatwaves (maximum daily temperatures > 36.6 °C) (Figure 1b); increase in tropical days (minimum daily temperatures > 20 °C), 3 to 6/decade (Figure 1c), and torrid days (minimum daily temperatures > 25 °C) (Figure 1b). Moreover, droughts point to a higher frequency because of lower relative humidity (Figure 1d) and lower annual volume of rainfall (i.e., Almería Airport, 1961–1990: 216 mm.; Málaga Airport: 583 mm.; Almería Airport, 1991–2020, 201 mm; Málaga Airport: 487 mm.); both values classify Málaga as a semiarid climate and Almería as an arid one. Floods and erosive processes from pouring rain also tend to be more frequent and hydric resources are decreasing [21,71,72]. Coastal phenomena and coastal losses are increasing [73,74]. Habitats and endangered species are increasingly suffering, with an increase of waterborne diseases predicted in the near future [75]. Finally, measuring anthropogenic gases allows their exhaustive control [76], which is vital to analyze the evolution of triggering factors for global warming. No significant trend has been identified regarding UVR, despite a slight trend towards decrease. These results agree with other estimations conducted for Andalusia by the Ministry of Ecological Transition (MITECO) [77], as well as with general estimates for the Mediterranean region by other official reports [78].

### 3.2. Ocular Pathologies Related to Climate Variables

All the pathologies described in chapter 2 are described in detail in Table S1, where each pathology is related to the climatic and environmental variables that affect them. Furthermore, in order to address this problem concisely, environment variables and potentially affected eye structures have been crossed in Table 1. Both the environmental values of Southern Spain and the most frequent ocular pathologies in that region have been considered.

### 3.3. Estimation of the Health Costs of the Most Frequent Ocular Pathologies Related to Climate Change in Southern Spain

Concerning Tractional Retinal Detachment (TRD), this is one of the most serious and costly pathologies, varying, depending on surgery type, from 1647 euros/patient for laser prophylaxis to 6690 euros/patient for vitrectomy [79]. The annual incidence of TRD in Spain is 1/10,000 [80], meaning 4700 cases/year.

Each heatwave increases the TRD risk by 2.47 times for people under age 75 years. In Spain, in 2020, the number of days per year in which heatwave thresholds are exceeded is twice as high as during the mid-1980s [81]. AEMET (Spanish State Meteorological Agency; http://www.aemet.es accessed on 9 January 2021) climate projections for the 21st century show an increase of about 20% in the number of warm days by the middle of the century; the frequency of extremely hot nights has increased tenfold from 1984 in the more populated Spanish cities [81]. Thus, for example, the threshold to consider a heatwave in Malaga city (Southern Spain) is 36.6 °C [82], which has been exceeded on 11 days during the summer of 2019 and seven during the summer of 2020. This implies an increase in TRD risk for people under age 75 years calculated at 17.3% only during that one summer. The trend identified from REDIAM data is a doubling of heatwave days from 1992 to 2020 (from 4 to 8 days) (Figure 1B).

Concerning Age-Related Macular Degeneration (AMD), people exposed to summer sun >5 h/day during adolescence and 30 years of age, at the initial examination, were at greater risk of developing increased macular pigment and early AMD (OR 2.14; CI = 95%, 0.99–4.61; *p* = 0.05) 10 years earlier than people sun-exposed <2 h/day during the same periods. Nevertheless, in patients reporting high summer sun exposure during their teenage years and 30 years old, the use of hats and sunglasses for half of those periods were associated with a lower risk of soft drusen (Relative Risk (RR) = 0.55; CI = 95%, 0.33–0.9; *p* = 0.02) and epithelial depigmentation of retinal pigment (RR = 0.51; CI = 95%, 0.29–0.91; *p* = 0.02) [83].

The progressive recovery of the ozone layer from international action on chlorine-fluorocarbon gases has achieved the maintenance or even a slight reduction in the average UVR (UV index) [84,85]. However, the trend of decreasing relative humidity due to increasing average temperatures aggressively increases UVR due to the lower radiative forcing (higher penetration) of this wavelength because of less filtering from water aerosols; moreover, air pollution also enhances the UVR penetration [86,87].

In 2006, it was estimated that by 2015, 400,000 Spanish inhabitants would suffer AMD, and more than one million could be at risk [50]. This reached 700,000 people in 2016 (1.5% of the Spanish population), almost twice as many as expected. Other factors which increase the risk of AMD are malnutrition and undernourishment [88]. Adverse and extreme climate events all over Spain are linked to a subsequent increase in agricultural, fruit and vegetable prices, which are essential to prevent the development of pathologies such as AMD and Glaucoma [89,90]. The annual burden of AMD is 8300 euros/patient, and half of this (48%) must be borne by the patients and their families.

Regarding glaucoma, direct costs exceed 612 euros/patient/year in Spain, whilst costs due to productivity losses reach 1946 euros, making a total cost of 2558 euros/patient/year [58,91]. Between 2011 and 2016 glaucoma cases in Spain increased by a factor of 3.77 (from 206,806 to 779,221 euros) [92]. From 1990 to 2015, the number of disability-adjusted life years (DALY) and the age-standardized DALY rate for glaucoma increased by 122% and 15%, respectively; in addition, glaucoma was associated positively with national levels of UVR and PM_2.5_. Besides, lower socio-economic level, advanced age, female gender, higher UVR (increasing the incidence by 11% depending on the number of hours outdoors) and higher air pollution level was significantly associated with a higher frequency of glaucoma [93]. Thus, as in AMD, glaucoma is conditioned at its onset by oxidative stress, secondary to UVR (UVA-UVB) [94,95], and its worsening and progression are also enhanced by malnutrition and undernourishment [50].

Besides this, cataracts surgery is one of the most frequent surgical procedures in the world; it is estimated that >22 million people/year underwent surgery [96]. The association UVR–cataracts has long been studied [12,14,61,97], and an odds ratio has been estimated with a 1.1 to 2.5 times higher risk of cortical and sub-capsular cataracts with higher exposure to UVB radiation; however, the use of sun-glasses has an odds ratio of 0.62 for posterior sub-capsular cataracts, thus protecting against the development of this type of cataracts.

**Figure 2:**
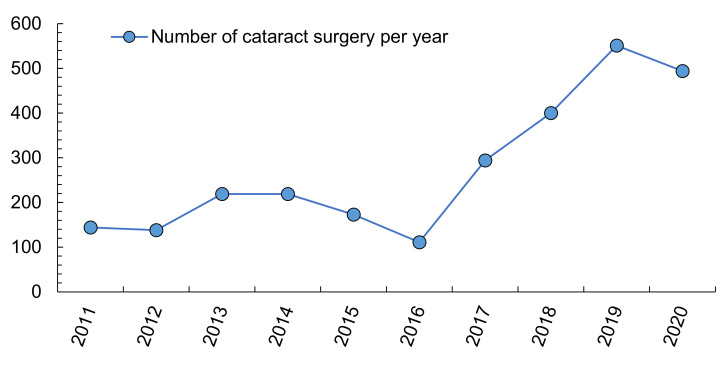
Cataract surgeries in a local hospital of Southern Spain (Axarquía Hospital). Own elaboration from Servicio Andaluz de Salud, 2021 [100].

The average cost of a cataract operation in Spain ranges between 910 and 1541 euros [58]. Because the number of cataract operations increased by 50% between 2004 and 2013 in Spain (almost 307,000 procedures) [98], a period in which the Spanish population grew by only 10% and elderly people by only 2% compared to 2004 [99], and because local data show a greater increase recently (Figure 2), it is necessary to address predisposing factors, since health care spending tends to increase year after year.

In the case of infectious diseases such as Herpes Zoster (VHZ), there is a 14% increased risk of outbreaks, especially in males, with overexposure to UVR, due to the immunosuppression it produces [8,101]. The average cost of treatment varies between 301.5 and 916.7 euros/outbreak, depending on its complication [102], with an estimated incidence of 60,000 cases/year [103].

Concerning Ocular Herpes Simplex (VHS), the risk of appearance and relapse increases, not so much because of the number of hours of outdoor activities, which could be a protection factor with a UV index < 4, but when these activities are carried out with a UV index > 4. Thus, a UV index > 4 increases the risk of a VHS outbreak by 33% [7]. In Southern Spain, the UV index > 4 is usually present from April to October [86]. The VHS incidence in Spain from 2000 to 2008 grew by 40% [104,105]. A global cost between 2033 and 2366 euros/outbreak/patient has been estimated, including costs for the medical procedure, sick leave, treatment and use of facilities. Between 2011 and 2017 116,000 cases/year were recorded, with a trend towards increase, only interrupted in 2017 [106].

Regarding auto-immune systemic diseases with ocular impact (non-infectious uveitis), it is necessary to highlight that environmental pollutants are one of the main triggering factors for its development, emphasizing especially:

(a) In Rheumatoid Arthritis (RA), genetic factors explain <50% of the risk of developing this disease; however, a 31% increase in risk has been identified in people living within <50 m from the main road (with traffic and, therefore, intense pollution) [107]. In Spain alone, RA accounted for a €1.12 billion/year burden of necessary health care, indirect costs and associated sick leave [108]. Mitigating the pollutant factor could save the Spanish State 350 million (M) euros, as well as undoubtedly reducing the suffering caused by this type of disease.
(b) In Ankylosing Spondylitis (AS), a >60% correlation with pollutant particles (PM_2.5_) has been found: a prolonged exposure leads to worsening in the control of the inflammatory outbreak of this disease [109]. An average cost to address AS in Spain of 11,462 euros/patient/year has been estimated, including direct costs and productivity losses [110]. In 2017, 1.9% of Spanish people suffered from this disease (900,000 people), implying an annual average cost of €10.3 billion of the Spanish Gross Domestic Product (GDP). Such a cost could be reduced by €6 billion just by controlling environmental factors.
(c) Pollutants determine an association with inflammatory outbreaks of Multiple Sclerosis (MS). Thus, PM_10_ particles were 8% associated with an increase of relapse during the cold season, whilst ozone was 16% associated during the hot season (therefore, ozone is pathogenically triggered by high temperatures) [111,112]. Around 50,000 people in Spain suffer from this disease, whose average cost is 30,000 euros/patient/year; this implies a global burden of €1.4 billion/year [113], added to the severe disability which it involves. 112 to 224 million euros/year could be saved by controlling pollutants alone, without taking into account the benefit to be gained by controlling high temperatures and, thus, avoiding the exacerbation suffered by MS patients (Uhthoff phenomenon) [112,114].
(d) Inflammatory Bowel Diseases (IBD) include Crohn’s disease (CD), Ulcerative Colitis (UC) and Non-classifiable IBD. Their estimated prevalence is 0.3% of the European population, showing an increasing incidence of 176,000 cases per year. It has been calculated that 2.5–3 million people in Europe suffer from IBD. This implies a very high total annual cost of health care. Based on an average of diagnosed patients in 2010 throughout 28 European medical centers during the first year after diagnosis, a total cost of 5942 euros/patient for CD, 2753 euros/patient for UC, and 2898 euros/patient for non-classifiable IBD was calculated. Furthermore, each day of heatwave increases by 4.6% the outbreak risk for IBD [115,116], and thus the derived visual impairment. For example, Málaga city (Southern Spain) suffered 11 heatwave days only in 2019; therefore, the outbreak risk of IBD was increased by 50.6%, adding to the high health care burden and personal suffering which this implies.
(e) The prevalence of Systemic Lupus Erythematosus (SLE) in Spain is 9/10,000 inhabitants [117]. The patient/year cost ranges between 3604 and 5968 euros, according to the severity [118]. Relating to prevalence and cost per patient, the control of this disease involves a €201 million burden in Spain. Concerning environmental parameters, SLE outbreaks are twice as likely to occur in sun-exposed workers fin a year, and 7.9 times more likely to develop if they suffer sunburn from intense exposure to the sun [119].
(f) Sarcoidosis is a disease with high prevalence in Spain (1/1000 inhabitants) [120]. Although there is no information about the cost of its treatment, data from the USA points out that insurance companies spend 19,714 dollars/patient/year among direct costs of medical attention and secondary costs because of sickness absences [121]. Extrapolating this data to Spain, the disease costs would be equivalent to almost €784 million/year. The relation between Sarcoidosis and environmental factors is essential, given that its pathogenesis is characterized by exposure to dust and both natural and urban pollution (PM_10_ and PM_2.5_), together with dryness and high temperatures [122]. Southern Spain records more than 20 events per year with Saharan dust advection from May to September [123]. There are no numerical studies on the evolution of this disease in Spain, but researches from the Midwest of the USA (where dust storms are frequent) reveal that the prevalence doubled between 1995 and 2010, without a relationship to an increase in the population [124]. Extrapolating these data to Spain, a higher frequency of dust storms related to higher dryness and advection from Africa could involve doubling of the health care costs related to Sarcoidosis.

The estimated costs of dealing with autoimmune diseases in the USA, where it has been studied more than in Europe, are over $100 billion, an underestimate since the eight most common autoimmune diseases alone account for between $51.8 and $70.6 billion/year [125]. Moreover, the chronic pain state and the disability to make changes in the mood of patients, sleeping disorders, and tiredness impact their social and working life [108].

The climate change-related eye diseases selected above represent an annual cost of between 22.63 and 23.31 billion euros, that is, between 1.9% and 1.95% of national Spanish GDP (Table 2). 36.5% of this figure can be attributed to climate change variables (0.7% GDP; between €8.26 and €8.51 billion). Mitigation measures centered on afforestation and greenhouse gases (GG) control involve a cost of €13.15 billion (1.1% GDP), but the expected benefit due to energy saving reaches between €16.5 and €25.7 billion (1.38–2.15% GDP). Thus, even with the least optimistic forecast, 0.28% GDP would be saved, adding the progressive reduction by 0.7% of GDP due to health care cost in ocular diseases partially attributable to climate change, and the intangible benefits of wellbeing and life-quality that reducing life-limiting illness implies (Supplementary Material Table S2). In any case, the cost of controlling GG would have benefits for any disease exacerbated by environmental variables related to climate change, and not just eye disease.

(1) Because of the sustained increase of CO_2_ concentration in Spain [126]
(2) The trend in Andalucía has been practically stable since 2008, whilst the average of Spain reveals a clear decrease, showing values below the average of Andalucía [127]
(3) In Andalucía during the last decade, the WHO criteria have been permanently broken (>100 µg/m^3^); there is no aggregated evolutionary data [128]
(4) Considering just the costs of afforestation-reforestation and GG control, and ignoring the benefits of energy-saving

**Figure 3:**
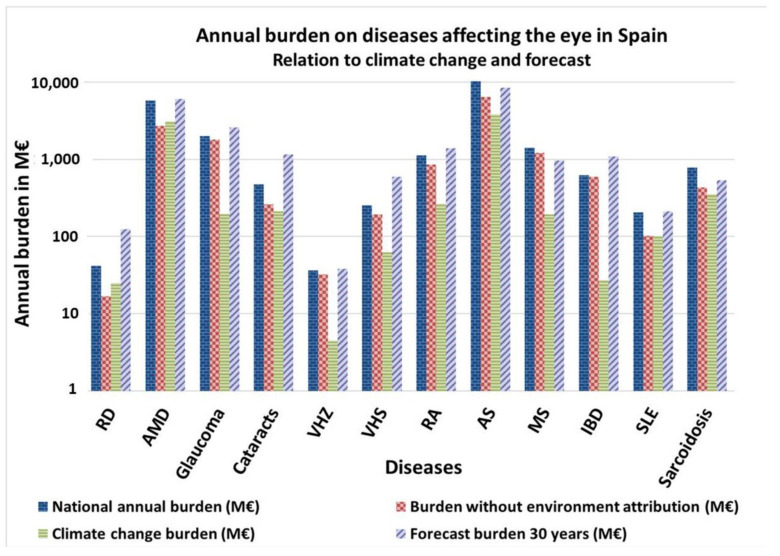
Annual burden of diseases affecting ocular structures in Spain. Relation to climate change and 30 years forecast. Own elaboration. RD = Retinal detachment. AMD = Age-Associated Macular Degeneration. HZ = Herpes Zoster. HS = Herpes Simplex. RA= Rheumatoid Arthritis. AS = Ankylosing Spondylitis. MS = Multiple Sclerosis. IBD = Inflammatory Bowel diseases; SLE = Systemic Lupus Erythematosus.

RD, AMD and SLE are the ocular impairments most affected by climate change, the cost of addressing them doubling due to environmental factors (Figure 3). Concerning the 30 years forecast, if the actual trend continues without intervention, diseases such as RD, AMD, Glaucoma, Cataracts, VHZ, VHS, RA, IBD and SLE will increase their incidence, and, subsequently, their associated burden.

## 4. Discussion. Challenges and Topics to Be Further Discussed

There is a wide and varied bibliography about the climate–ocular health relationship. Global warming behaves like a chain reaction that ominously affects eye health [129]. However, it is uncommon to find interdisciplinary studies where ophthalmologists and environment specialists work together. In the context of climate change, we start from the premise that if adequate management can mitigate the effects of enhanced climatic variables [130], it can consequently mitigate health problems. This study highlights the possibility of preventing an increase in eye diseases and their associated health costs through sustainability, taking into account that this aim can only be reached through environmental, social and economic balance [131], besides cultural factors.

The WHO [132] reports that >28% of the world’s population is visually impaired and almost half of this could be prevented. The same report highlights that “*…while the median out-of-pocket spending on health represents <20% of total health spending in high-income countries, it accounts for >40% in low-income countries*”. Additionally, rural areas of many countries have low coverage in ocular health care (childhood myopia, cataract) whilst urban lifestyle leads to an increase in myopia and diabetes complications (i.e., diabetic retinopathy).

Reducing global warming needs a reduction in GG emission, and other actions related to afforestation, reforestation and changes in crop management, given that the latter measures favor carbon sinks; furthermore, in urban environments, modulating temperature means controlling emissions of ozone, PM_10_ and PM_2.5_, as well as other pollutants; there is also a need to change the albedo of buildings and increase green spaces. The increase in radiation (especially UV) can be stopped if the increase in temperature is effectively controlled.

The benefit of the intervention in climate change variables could be appreciated via the reduction of related ocular pathologies. This would represent an important decrease in human morbidity and a significant reduction of the health care burden needed to address it. A carbon sink has been defined as any process or mechanism that removes a greenhouse gas, an aerosol or a precursor of greenhouse gas from the atmosphere. A given reservoir can be an atmospheric carbon sink if, during a given time interval, more carbon enters than leaves [133]. Similarly, any process or mechanism that eliminates or reduces a harmful effect or environmental health risk can be defined as a disease sink. In this way, this new concept of disease sink can also be understood as any action in favor of climate change mitigation. As was mentioned, the effects of climate change have direct impacts on the incidence or morbidity of diseases. Consequently, mitigation measures must involve a health gain, as a disease sink.

The real financial cost has two components: direct (health-related) and indirect (production losses, informal care and losses in wellbeing). It has been estimated that the total cost of health care for visual impairment in 2010 reached $1100 billion globally, excluding uncorrected refractive errors; the global financial cost reached $2954 billion. These costs include inpatients care, treatment, general eye services, and community and elderly people care, among others. In addition, associated costs of traumatism due to the visual impairment have been included [134].

Mitigation strategies look to reduce the net emissions to the atmosphere of long-term GG, which is the “meal” of anthropogenic climate change [54,135,136] and also look for an increase in the natural systems to eliminate these GGs. This reduction can be reached by:

(1) Increasing the capacity of the carbon sink by means of afforestation and reforestation. From 1990 to 2020, the world lost 420 million (M) hectares (ha) of forest area; 80 M ha of these corresponded to primary woods [137]. Andalucía is one of the three Spanish communities which are developing an emission compensation system using forest projects [138]. The economic cost for one of these projects is 229,351.5 euros for 63.42 ha, and it is expected to capture 16,653 tCO_2_ in 30 years, that is, to assimilate 262.6 tCO_2_ per forestry hectare. According to the actual GG emission in Andalucía, where there was an annual production of 51 M tons CO_2_eq in 2015, it would be necessary to invest €702.27 M to offset through forestry works the GG emissions, and afforest or reforest 194,211.7 ha. Obviously, current efforts are insufficient.
(2) Avoiding ocean acidification. In Andalucía, to avoid the acidification of the sea, and to control the waste of plastics, emissions of nitrogen fertilizers and untreated wastewater, it is essential to control overfishing and highly destructive fisheries to prevent the destruction of carbon sinks [55]. Furthermore, to stabilize emissions at around 450 ppm of CO_2_eq (recommended by IPCC), it would be necessary to reduce the annualized consumption growth rate by 0.06% per year during the 21st century [56]. While Global GDP (2019) is $87,698 billion [139], the efforts to achieve stabilization would be equivalent to $5.26 billion/year. It has been calculated that Spain would have to reduce its GDP by 1% by 2050 to comply with the limits set by the Kyoto Protocol, i.e., some 12.45 billion euros over 30 years [140].
(3) Promoting the transition of the actual energy generation (electricity) to systems of low carbon emissions. In Spain, the Ministry for Ecological Transition has proposed that 70% of the electric system in 2030 came from renewable energies. Investments in this sector, energy savings and renewable energies (which are cheaper) will allow the GDP to grow by 1.8% by 2030 compared to a scenario without actions: between €16.5 and €25.7 billion [141]. This figure compensates for the reduction in GDP indicated in the previous item.
(4) Increasing building insulation. Global warming makes predictable an increase in energy consumption for cooling buildings and homes. Current energy-efficient systems allow a building with solar protection, efficient ventilation and an insulated façade to save 38% in heating and 52% in cooling [142]. It is expected that the world energy consumption increase will average 57% between 2004 and 2030 [143]. Therefore, energy efficiency and near-zero energy homes will be essential because they have no additional costs [144].
(5) Ensuring that new buildings use more natural air and sunlight. Accordingly, the efficient design of buildings through ventilation with heat recovery, building skin insulation without thermal bridges and dynamic solar control using a blind system (passive house model), would obtain a fairly constant temperature throughout the year, without exceeding 25 °C in summer, i.e., higher energy efficiency [142].
(6) Holding back growth of energy demand: promoting private, public and collective transport, which would reduce around 25 Mt CO_2_ [145]; an energy culture based on saving and using more efficient and renewable energies; environmental tax to stimulate the change to non-wasteful production models [146].
(7) Stop playing with Nature [147]. As mentioned above, environmental intervention due to inadequate land management, often because of lack of knowledge, produces irreversible ecological cascades which, increasingly common, affect human beings tragically [148]. One probable origin of Covid19 disease is the uncontrolled alteration of ecosystems (contact with wild animals such as the bat *Rhinolophus affinis*, or the Malaysian pangolin, *Manis javanica*) [149] and its triggering due to pollution [150]. Other unfortunate examples of human interventions were the myxomatosis virus [151] and HIV, possibly from apes [152].

All these measures could be included in the new concept, disease sink, because they contribute to decreasing the effects of climate change, and therefore reduce the incidence and morbidity of diseases.

The estimates made throughout this research are limited to diseases affecting the eyes; they are just one example, taken from Southern Spain, of the impact of climate change on human health and the associated expenditure. Ocular diseases depend on environmental factors related to climate change by up to 36.5%, which implies an annual burden of 0.7% of the Spanish Gross Domestic Product.

Returning to the goals of this research, the literature consulted amply demonstrates the links between environmental factors related to climate change and eye diseases. Applying this fact to an arid and semiarid area such as Southern Spain, the analysis of environmental variables shows a high vulnerability in the population to suffer increasingly from eye diseases due to the impact of climate change: higher temperatures, more frequent droughts, forest fires, floods and erosive processes from pouring rain, depletion and pollution of water resources, coastal losses and coastal phenomena, habitat alteration, water-borne diseases and other altered variables like ground-level ozone, pollutant precursors of ground-level ozone, greenhouse gases, acidifying agents, PM_10_, PM_2.5_, persistent organic pollutants, benzene, aromatic polycyclic hydrocarbons, dioxins, and furans. In addition, Andalusia’s gross domestic product accounts for 13.33% of Spain’s GDP. In contrast, the Andalusian population accounts for 18.1% of the total Spanish population. This implies lower levels of income and resources for Andalusia, which is the third Spanish autonomous community with the lowest income per capita (19,658€ compared to a national average of 26,438€), only ahead of the autonomous cities of Ceuta and Melilla [53]. Increased risk due to the above mentioned environmental factors and reduced availability of resources contribute to poorer eye health. Exactly to what extent they influence this region should be the subject of future research.

Finally, we have estimated the health expenditure for the fight against these diseases as between 22.63 and 23.31 billion euros per year, as can be seen in Table 2. We have estimated that, on average, 36.5% of this expenditure is attributable to the increase in eye diseases due to environmental factors related to climate change. There is no exact data at the regional level, but the higher incidence of climate change effects estimated for southern Spain indicates that a significant share of this figure corresponds to this region. In an ideal scenario of mitigating the effects of climate change, considering only the costs of afforestation-reforestation and greenhouse gas control, and ignoring the benefits of energy savings, an investment of 13.15 billion euros would be required, but this value is useful for any disease exacerbated by environmental variables related to climate change: this would be the estimated price of disease sinks for Spain.

Adaptation to climate change, therefore, would imply a permanent annual increase in expenditure on eye health to try to mitigate the effects of this process. Mitigation, on the other hand, would imply a reduction in expenditure, as well as an improvement in the health and quality of life of the population. The verification of this fact will depend on the success of global and local measures to mitigate the effects of climate change, but our data suggest that adaptation to climate change involves permanently increasing costs, while mitigation could reduce these if successful.

## 5. Conclusions and Implications

The increase in eye diseases will have a strong economic and social impact if no mitigation measures are taken against climate change, as much evidence points to a link between weather and climate variables enhanced by climate change and eye-related diseases. Therefore, if proper management can mitigate the effects of climate change, consequently, this can reduce health problems. We propose a new concept: disease sink. By analogy with carbon sinks, a disease sink can be understood as any action to mitigate environmental factors exacerbated by climate change that reduces the incidence or morbidity of disease and consequently generates a health benefit. Producing disease sinks can also bring social and economic benefits resulting from improved environmental quality and savings in climate change adaptation measures. Our data and the scientific literature analysed indicate that southern Spain is highly vulnerable to the effects of climate change. Higher temperatures, more frequent torrential rainfall and droughts, fires, floods, coastal phenomena, alteration of water resources, alteration of habitats and poor pollution control are all elements that directly or indirectly exacerbate numerous eye diseases. At the national level, the estimated health expenditure for the fight against these ocular diseases alone is between 22.63 and 23.31 billion euros per year, with 36.5% of this figure attributed to environmental variables exacerbated by climate change. There is no exact data at the regional level, but the higher incidence of climate change effects estimated for Andalusia indicates that a significant part of this figure is for this region, one of the lowest per capita income regions in Spain.

It would be advisable for other medical specialities to conduct similar studies to emphasize the need to mitigate climate change, as the health care costs of non-intervention in climate change will probably be unaffordable. In addition, more frequent contacts between climate and environmental scientists and medical specialists for joint research would also be desirable. This fact is especially remarkable in those areas where the effects of climate change have the greatest impact: arid and semiarid zones.

In conclusion, medium-term mitigation and long-term reversal of the effects of climate change are essential for our health; adaptation is not an option, as it requires more funds, which not all countries or regions can afford. We hope that this research will guide researchers interested in the role of climate change concerning health in general and eye health in particular, and will help policymakers to redress the economic damage associated with a policy of non-intervention regarding climate change.

## Figures and Tables

**Table 1:** Relationship between environmental variables and potential eye damage.

Environmental Variable	Cornea, Sclera and Conjunctive	Glaucoma	Cataracts	Tumours, Iris, Choroid and Ciliary Body	Uv. Inf.	Uv. No inf.	Retina	AMD and Central Serous Choroidopathy
Rainfall	X				X	X	X	X
Temperatures	X	X			X	X	X	X
Humidity	X	X			X	X	X	X
Wind	X				X			
Insolation/UV Radiation	X	X	X	X	X	X	X	X
Air Pressure		X						
Sea level					X			X
Albedo			X			X	X	X
Ozone	X					X		
GG	X	X				X		
PM_10_ and PM_2.5_	X	X				X		
Other pollutants	X	X				X		
Other indirectly related factors (malnutrition, malnourishment, water consumption)	X	X			X			X

Own Elaboration. GG = Greenhouse Gases. Uv. Inf.: = Infectious uveitis. Uv No Inf.= Uveitis not infectious. AMD = Age-related Macular Degeneration.

**Table 2:** National Annual Costs of diseases involving eye structures, their attribution to climate change, expected evolution and mitigation costs.

Pathology	National Annual Costs (€M or €B)	Attribution to Climate Change	Attributable Increase to Extreme Events (%)	Estimated Increase	Investment for Mitigation (€B)
Retinal Detachment	€16.47–66.9 M	Heatwaves	147	Doubling in 30 years	
AMD	€5.81 B	UVR Summer sun	114	2% × Δ °C^−1^	
Glaucoma	€1.99 B	UVR PM_2.5_	11 Undetermined	Five-year tripling −0.59% year^−1^	
Cataracts	€ 473 M	UVR	10–150	Δ4.8% year^−1^	
Herpes zoster	€18–55 M	UVR	14	2% × Δ °C^−1^	
Herpes simplex	€235.83–274.46 M	UVR	33	2% × Δ °C^−1^; Δ4.4%year^−1^	
Rheumatoid Arthritis	€1.12 B	Pollution	31	0.8% year^−1^ (1)	
Ankylosing Spondylitis	€10.236 B	PM_2.5_	60 (outbreaks)	−0.59% year^−1^ in Spain; −0.17% year^−1^ in Andalucía (2)	
Multiple Sclerosis	€1.4 B	PM_10_ + cold Ozone + heat	8 (outbreaks) 16 (outbreaks)	−1.05% year^−1^ in Spain −0.57% year^−1^ in Andalucía 0.47% Year^−1^ according to WHO criteria (3).	
Inflammatory Bowel diseases	€391–844 M	Heatwaves	4.6 (outbreaks)	Doubling in 78 years	
Systemic Lupus Erythematosus	€153.5–254.2 M	Sun exposition	100 (outbreaks) 790 (sunburns)	2% × Δ °C^−1^	
Sarcoidosis	€784 M	PM_10_ PM_2.5_ Dryness (Δ1% decade^−1^) Rising temperatures (0.13–0.4 °C decade^−1^)	82 Undetermined Undetermined Undetermined	−1.05% year^−1^ −0.59% year^−1^	
Total Amount	€22.63–23.31 B		36.46 (€8.37 B)		13.15 (4)
% burden with respect National GDP (€1194 B)	1.9–1.95		0.75		1.1
Benefits of changing energy model					16.5–25.7
% saving of National GDP (€1194 B)					1.47–2.29

Own elaboration.

## Data Availability

Source data are cited in the article. Supplementary Materials Tables S1 and S2 are Excel spreadsheets with processed data from REDIAM (Supplementary Material Table S1) and the different cost calculations by pathology applied from the referenced literature and Andalusian Health Service data (Supplementary Material Table S2). https://drive.google.com/file/d/1LvqRyH_DV83waommEG5wdYI0ptbrQXWl/view?usp=sharing; https://drive.google.com/file/d/1q_eTEw5HAvXm5aWU_s42eS9-Kce63ojS/view?usp=sharing (accessed on 4 July 2021).

## Supplementary Materials

The following are available online at https://www.mdpi.com/article/10.3390/ijerph18137197/s1, Table S1: Pathologies with ocular involvement related to environmental variables, Table S2: Trends of environmental variables in Southern Spain. Table S1 and S2: https://drive.google.com/file/d/1sgHTDZxsekH8O7v8sL6gJVE9GtpWa6k2/view?usp=sharing (accessed on 4 July 2021). References [153,154,155,156,157,158,159,160,161,162,163,164,165,166,167,168,169,170,171,172,173,174,175,176,177,178,179,180,181,182,183,184,185,186,187,188,189,190,191,192,193,194,195,196,197,198,199,200,201,202,203,204,205,206,207,208,209,210,211,212,213,214,215,216,217,218,219,220,221,222,223,224,225,226,227,228,229,230,231,232,233,234,235,236,237,238,239,240,241,242,243,244,245,246,247,248,249,250,251,252,253,254,255,256,257,258] are cited in the Supplementary Materials.
