# Supplementary material for: Impact of Climate Change on Eye Diseases and Associated Economical Costs
Source: Int J Environ Res Public Health. 2021 Jul 5;18(13):7197. doi: 10.3390/ijerph18137197 (PMC8297364; doi:10.3390/ijerph18137197)
Supplement: Supplementary file 1 [file ijerph-18-07197-s001.zip › ijerph-1252216-supplementary.pdf]

**Table S1.** Pathologies with ocular involvement related to environmental variables.

| **1. Corneal, Scleral and Conjunctival pathologies** | **Related environmental variables** |
|---|---|
| **Allergic keratoconjunctivitis (A)**. Its frequency increase with high levels of $NO_2$ and ozone, but also with higher temperatures and humidity. In addition, it has been documented an increase of allergic patients because of earlier and longer pollination season. Finally, the damage in conjunctive because of organophosphate pesticides and organochlorine herbicides dysregulates the conjunctive immune system's defenses, enabling allergic reactions to environmental pollen [13 66 174 175 176]. | $NO_2$<br>Ozone<br>Higher temperatures<br>Humidity.<br>Longer pollination season<br>Organophosphate pesticides and organochlorine herbicides |
| **Marginal keratitis (A)**. It is a hypersensitivity reaction to exotoxins and proteins of the staphylococcus cell wall that may be related to chemical pollutants [66]. | Chemical pollutants |
| **Dry keratitis** ("dry eyes") **and Photo-keratitis (A)**. They have been related to prolonged exposure to UVR, but, in the case of dry keratitis, also to pollutants and weather changes [13 14 176 177 178]. | UVR<br>Pollutants<br>Weather changes |
| **Chronic Episcleritis (A)**. It is related sometimes with rheumatoid arthritis and other connective tissue diseases, which are triggered by several chemical pollutants and foods [66]. | Chemical pollutants<br>Foods |
| **Corneal metaplasia (A)**. Several authors refer the "spheroidal degeneration" (climatic drops keratopathy) as a pathology related to the exposure to environmental irritants, such as evaporation, micro-traumas because of dust and sand have blown by the wind, and mainly, UV radiation [14 57 177 179]. | Evaporation<br>Wind<br>UVR |
| **Pterygium (A)**. It has been documented its increase of incidence and relapse; in addition, it has been highlighted that its outbreaks increase because of heat waves and chronical damage due to ultraviolet radiation [10 12 14 51]. | Heatwaves<br>UVR |
| **Corneal and conjunctival Ocular Herpes simplex and Herpes Zoster (B)**. It has been registered an increasing frequency consistent with an increase of UVR, both intra-year and yearly because a higher insolation [7 8 13 101 180]. | UVR<br>Higher insolation |
| **Viral keratoconjunctivitis (B)**. There is hardly a reference about its relation with climatic or environmental factors despite is a common pathology. Only an old research highlights a higher outbreaks frequency around the extra-tropical world during the autumn [181]. | Higher frequency during autumn |
| **Accidental corneal fungal injuries (B)**. Fungal ulcers are more frequent in warm climates. It has been observed a sustained increase of fungal keratitis at the same time that a rising of minimum temperatures, and a correlation between the rising of temperatures, the resulting rising of carbon dioxide, and an increase of the incidence of kerato-mycosis [182 183 184]. | Higher in warm climates<br>Rising minimum temperatures<br>Rising $CO_2$ |

| | |
|---|---|
| **Infectious corneal injuries no fungal by contact lens or trauma (B)**. These are ulcers and keratitis because of acanthamoeba and pseudomonas presents in water for human consumption (i.e., polluted by pouring rain) and it can be expected an upturn in Andalucía. *Pseudomonas aeruginosa* has also been related to warm seasons, especially summer and autumn; in addition, it shows an upward year-on-year trend [13 61 185]. | Water consumption<br>Contaminated water<br>Rainfall<br>Warm seasons |
| **Epidermoid neoplasia of ocular surface (C).** Several authors point that pathologies such as Conjunctival Epithelial Dysplasia, Carcinoma in situ, or Corneal and Conjunctive Epidermoid Carcinoma are caused by an excess of UV radiation [13 177]. | UVR<br>Warm regions |
| **Basal Cell Carcinoma of Eyelid (C)**. As in the previous group of diseases, it has been pointed out its relation with an excess of UV radiation [13 14]. | UVR |
| **2. Glaucoma** | **Related environmental variables** |
| **Acute glaucoma (D).** Its frequency increase with variable and cloudy weather, and also with increasing pressure, humidity and temperature [186]. | Cloudy weather<br>Increasing pressure<br>Increasing humidity<br>Increasing temperature |
| **Chronic Open-angle glaucoma (E)**. Its development has been related to urban pollutants, as well as tobacco smoke. Others authors point to the incidence of UVA and UVB radiation, which causes DNA oxidative damage, elevating the intraocular pressure. In addition, it has been highlighted a positive association between glaucoma and national levels of UVR and $PM_{2,5}$ [42 43 93 95]. | Urban pollutants<br>Tobacco smoke<br>UVR<br>$PM_{2,5}$ |
| **3. Cataracts.** | **Related environmental variables** |
| **Early cortical and subcapsular cataract (F).** Cortical cataracts have been studied as a consequence of exposition to UV-B radiation. Furthermore, they have been related both cortical and posterior subcapsular cataracts with the increasing UVR and the depletion of the ozone layer [12 14 61]. | UVB<br>UVR (in general)<br>Depletion of the ozone layer |
| **Pseudoexfoliation syndrome (G).** It is a cataract with pseudoexfoliation material that uses to be at high surgical risk and high increasing of intraocular pressure. It is appearing with an increase of a frequent sun exposition, and also uses to be frequent both in places of high altitude and higher radiation (higher albedo due to snow). However, it is a protector factor the increasing of minimum winter and summer temperatures [187]. | Sun exposition<br>High altitude<br>High radiation<br>Albedo<br>Increasing the minimum temperatures (protection factor) |
| **4. Tumors processes in the choroid, iris and ciliary body** | **Related environmental variables** |
| **Uveal Melanoma (H)** is the main disease of this group related to environmental factors. Several authors relate it with exposure to UVB radiation [12 60 61]. | UVB |

| 5. Uveitic processes | Related environmental variables |
|---|---|
| **Infectious uveitis (I)** | |
| **Toxoplasmosis (a)**. Some authors point a clear increasing trend for the incidence of Toxoplasmosis because of temperatures, on account of a higher facility for the survival of parasites and its vector transmission. In addition, heavy rain improves the parasite flow to drinking waters; thus, due to its chlorine resistance, may result in outbreaks of epidemic toxoplasmosis. The more frequent relationship of human beings with animals is also responsible for its higher frequency. Finally, it has been highlighted the relation between Toxoplasmosis and Fuchs's heterochromic uveitis. Ocular toxoplasmosis represents 20-60% of all posterior uveitis [62 188 189]. | Temperatures<br>Heavy rain<br>Drinking water<br>Relationship with animals |
| **Tuberculosis (b)**. Despite it was expectable a decreasing of this disease thanks to vaccines and modern anti-microbial treatments, it is yet increasing because of human displacements in addition to bacterial resistance due to indiscriminate use of anti-microbial, which it has been applied to control zoonosis, and natural environment invasions by human beings; this allows for increased contact with pathogens and enhance adaptation of pathogens to the human environment. Ocular tuberculosis presents granulomatous anterior uveitis, with frequent vitritis, choroidal granulomas, choroiditis without tubercular, and retinal vasculitis (related with Eales' disease) [65 190 191 192 193]. | Bacterial resistance<br>Natural environment invasions |
| ***Campylobacter* (c)**. It has been related a dangerous increase of this bacteria and the changes in temperatures and rainfalls. This germen is related to Reiter's syndrome, which presents conjunctivitis and uveitis in ocular structures [13 194]. | Temperatures<br>Rainfall |
| ***Chlamydia pneumoniae* (d).** It is highly sensitive to increase because of all climate change variable: temperatures, pouring rain, UVR, wind, environmental pollutants, drought, low environmental humidity. *Chlamydia pneumonia* causes a respiratory infection which may be previous to reactive arthritis, appearing in addition to conjunctivitis, uveitis and episcleritis [195 196]. | Temperatures<br>Pouring rain<br>UVR<br>Wind<br>Environmental pollutants<br>Droughts<br>Low humidity |
| **Ocular herpes simplex and zoster (e)**. It has been highlighted an increase of infectious uveitis because of several types of herpes, that are the most frequent etiological agent of uveitis. Its relation with UVR and temperatures has been mentioned in the previous heading; however, in this case, it is related to anterior uveitis, acute retinal necrosis, Fuchs' heterochromic uveitis and Posner-Schlossman syndrome [68 197]. | UVR<br>Temperatures |
| **West Nile Fever (f)**. It is considered as an endemic-epidemic zoonosis in Europe, and it has an increasing incidence. It has a direct relation with pouring rain and warmer temperatures, which improve the vector | Pouring rain<br>Warmer temperatures |

| | |
|---|---|
| (*Culex pipiens* mosquito). It causes a typical multifocal chorioretinitis with injuries which follow up the course of retinal nervous fibers. Others clinical manifestations include anterior uveitis, vasculitis, optical neuritis, sixth par paralysis, nystagmus and congenital chorioretinal scaring [29,30,198]. | |
| **Borreliosis (g)**. An expansion of this disease has been provided to zones previously cool because of an increase of minimum temperatures; this may enhance the presence of ticks (*Ixodes scapularis*) where previously it was not present, because the borreliosis (Lyme's disease) is transmitted by this vector-borne. Lyme's disease produces anterior uveitis (granulomatous or not), intermedial uveitis (more frequent), and includes multifocal choroiditis, vasculitis and neuro-retinitis [199,200,201,202]. | Higher minimum temperatures |
| **Rickettsiosis (h)**. A recent increase in Mediterranean Spotted Fever (due to *Rickettsia conorii*) has been detected in mediterranean areas of Iberian Peninsula, coinciding with an increase in temperatures, especially in summer, and a decrease in rainfalls. This disease is transmitted by the tick *Rhipicephalus sanguineus*. Ophthalmologically, it shows conjunctivitis, anterior uveitis, retinitis, serous retinal detachment, optic neuropathy, vasculitis and panuveitis [203,204,205]. | Higher temperatures<br>Lower rainfalls |
| **Shigellosis (i)**. It has been found a seasonal relation between outbreaks of shigellosis and two climate variables: temperatures and rainfall; moreover, it has been added to those the increase of humidity. Thus, it has been detected in Spain a trend to increase. This disease may provoke reactive arthritis (Reiter's syndrome) in association with HLA B27 antigen; in addition, it causes conjunctivitis, uveitis and episcleritis [196,206,207,208,209]. | Temperatures<br>Rainfall<br>Increasing humidity |
| **Salmonellosis (j)**. It has been highlighted an increase of this infection related to an increasing of temperatures. Moreover, it has been related specifically extreme temperatures and rainfall with salmonellosis outbreaks, especially in coastal areas, because of flooding and contamination of drinking water. In particular, it has been detected in Spain a continuous increasing of this disease, which is related to reactive arthritis (Reiter's syndrome) in association in a 75% with HLA B27 antigen; it produces conjunctivitis, uveitis and episcleritis [62,63,196,208,209,210]. | Higher temperatures<br>Extreme temperatures<br>Extreme rainfall<br>Floods<br>Contamination of drinking Water |
| **Uveitis not infectious associated to systemic diseases (J)** | |
| **Rheumatoid arthritis (k)**. Its outbreaks have been related to environmental factors such as pollutants and occupational factors related to the use of mineral oils, upholstery, concrete works, hairdressers. It causes scleritis and sporadic uveitis [107,211,212]. | Pollutants |
| **Ankylosing spondylitis (l)**. This autoimmune disease is highly related to HLA B27+ antigen, whose uveitis processes are increasing lately. In addition, the worsening of its symptoms has been highly related to | Pollutants<br>$PM_{2.5}$<br>Foods |

| | |
|---|---|
| pollutants, especially $PM_{2.5}$, and moderately with a highly saturated fat diet. It causes anterior uveitis [65 109 213]. | |
| **Sarcoidosis (m)**. In the rural world, it has been related to livestock farms and sawmills, perhaps due to an infectious factor (*Mycobacterium* ssp, Treponema, Herpes simplex, Histoplasmosis). In addition, it also has been related to storm dust, and, in general, to dust exposition. Dust is common both in dry areas and droughts and in urban areas. Sarcoidosis has been related to seasonality (winter and, especially, summer), and the uveitis uses to be one symptom which allows an earlier diagnose; furthermore, its higher frequency in summer is related with higher temperatures, dryness and light intensity. A final key factor may be the hypersensitivity to talcum, beryllium, timber and silica dust, as well as mold, all the possible causes of granulomatous uveitis such as the sarcoidosis [66 122 214 215]. | Livestock farms<br>Sawmills<br>Storm dust<br>Dust exposition<br>Dry areas<br>Droughts<br>Urban areas<br>Summertime<br>Higher temperatures<br>Light intensity<br>Pollutants |
| **Multiple Sclerosis (n)**. Some authors point to a relation between relapses of multiple sclerosis and exposition to ozone, $NO_2$ and $PM_{10}$; these authors emphasize especially ozone, high temperatures and relapses of this disease, which cause anterior and intermedia uveitis, in addition to periphlebitis [111 216]. | Ozone<br>$NO_2$<br>$PM_{10}$<br>Higher temperatures |
| **Inflammatory bowel disease (Crohn and ulcer colitis) (o)**. It shows higher morbidity incidence and relapses during heat waves. This disease causes anterior uveitis [115 217]. | Heatwaves |
| **Behçet's disease (p)**. There is evidence about its relation with pollutants, in addition to some viral diseases such as Herpes simplex and a genetic predisposition (related to HLA B51 antigen), which may develop ocular and articular damage. Therefore, this disease depends directly on pollutants, and indirectly on solar radiation (which triggers Herpes simplex). Others authors point to weather changes as a related environmental factor. Ocular Behçet produces hypopyon, vitritis, necrotizing occlusive vasculitis, and often, panuveitis [218 219 220]. | Pollutants<br>Solar radiation<br>Weather changes |
| **Giant cells arteritis (Horton's disease) (q)**. It has a higher frequency in the metropolis. It also has been related to infectious diseases as Herpes simplex and Zoster, Chlamydia, *Mycoplasma pneumoniae*, Parvovirus B19 and Epstein-Barr and, therefore, with its predisposing factors. Its ocular symptoms include anterior, intermedia and posterior uveitis, panuveitis, cystoid chronic macular oedema, and, especially, ischemic optic neuropathy [221 222 223 224]. | Urban environment<br>Environmental factors of several infectious diseases |
| **Necrotizing systemic vasculitis (r)**. This includes several diseases such as polyarteritis nodosa, granulomatosis with polyangiitis, Wegener's granulomatosis, microscopic polyangiitis and Churg-Strauss syndrome. Some pollutants such as silica, asbestos and several infections (bacterial endocarditis, hepatitis C virus) are related to the outbreak of this disease. Others authors point to dust, livestock, heavy metals and dissolvers. | Pollutants<br>Dust<br>Livestock<br>Trace elements<br>Dissolvers |

| | |
|---|---|
| This disease causes acute anterior uveitis and hemorrhagic, exudative and occlusive retinal vasculitis [225 226 227 228]. | |
| **Connective tissue diseases (K)** | |
| **Systemic Lupus Erythematosus (s)**. Some authors have pointed out a relation between retarded triggering of this disease and long exposure to UV radiation. Others, on the other hand, have found an increased risk of developing and worsening Lupus related to sun exposure, solvents and silica (pottery and masonry workers). In addition, it has been highlighted a synergic action between UV radiation exposure and Cytomegalovirus for the 52-kD/Ro antigen expression, related in a 50% with Lupus. The number of ocular structures affected by this disease is wide: episcleritis, scleritis, anterior uveitis, occlusive retinal vasculitis, and serous retinal detachment [57 119 229 230 231 232]. | UVR<br>Sunlight<br>Dissolvers<br>Silica |
| **Dermatomyositis (t)**. A triggering of its outbreaks have been seen during April and May, in addition to its association with the pathogens Borrelia and Toxoplasma, mentioned above under the heading of infectious uveitis. This disease also has been related to maternal exposure to powdered chalk, maternal smoking, second-hand smoke, concentrations of carbon monoxide and, in general, inhaled pollutants. Other authors point to sun exposure, especially UVB radiation. The Dermatomyositis ocular manifestations include hypertensive anterior uveitis, retinal thrombophlebitis and cottony exudates [233 234 235]. | Springtime<br>Maternal exposure to:<br>-Powdered Chalk<br>-Smoke<br>-Carbon monoxide<br>-Inhaled pollutants<br>Sun exposure<br>UVB |
| **Uveitis just as an ocular disease -without associated systemic pathologies- (L)** | |
| **Fuchs' heterochromic Uveitis (u)**. It has multifactorial etiology, given that it is related to toxoplasmosis associated-diseases, which increase with temperature and heavy rain. It is also related to Cytomegalovirus, Herpes simplex and Chikungunya disease, virus all them which tend to increase with global warming. Only its relation with congenital rubella syndrome tends to decrease because of vaccination [188 189 236]. | Higher temperatures<br>Heavy rain |
| **Posner-Schlossman syndrome (v)**. It is also related to Cytomegalovirus and Herpes simplex, whose livelihood increases when subjected to UVR. Others authors points that outbreaks of Posner-Schlossman are favored by UVR exposure. This syndrome uses to show unilateral acute hypertension and anterior uveitis [57 237 238 239 240]. | UVR |
| **Intermedia uveitis and pars planitis (w)**. They have been related to sclerosis multiple and smoking habit, in addition to Herpes simplex, Human Herpes type 6, Varicella-Zoster and Epstein-Barr, and, therefore, their predisposing factors. However, D vitamin (which actives with UVR) may be a protective factor. Others authors point to *Chlamydia pneumoniae* as an agent involved in this uveitis, because of the improvement of sclerosis multiple when applied antibiotic treatment against *Chlamydia*, and, thus, the improvement of pars planitis. It should | Smoking habit<br>Environmental factors of several infectious diseases<br>Environmental factors which trigger *Chlamydia pneumoniae* |

| | |
|---|---|
| be remembered that *Chlamydia pneumoniae* is triggered by almost all climate change variables [195 241 242 243]. | |
| **Birdshot Choroidopathy (x)**. Related in 95% of cases to HLA A29 antigen, it has a poor visual prognosis; it is related to allergic problems and smokers. Its ocular symptoms involve retinal generalized bilateral vasculitis, with multiple hypopigmented and dispersed chorioretinal injuries [57 227]. | Pollen<br>Smoking habit |
| **Vogt-Koyanagi-Harada syndrome (VKH) (y).** Despite a genetic predisposition (HLA-DRB1*04 allele), there seems to be a viral trigger, such as Cytomegalovirus and Epstein-Barr, virus dependent on UVR exposure to increase its incidence. The variants of this syndrome may show bilateral anterior uveitis, exudative retinal detachment, or only retinal affectation [244 245]. | UVR |
| **White dots syndromes (z)**: It can have two variants:<br>**i) Acute posterior multifocal placoid epitheliopathy**. It has been related to viral processes and relapsed hypersensitivity reactions due to viral, fungal, bacterial and parasitic infections, such as tuberculosis, schistosomiasis, streptococcus, meningococcus, influenza, mumps, hepatitis B or dengue. Other authors point to its relation with Lyme disease, sarcoidosis, Wegener's disease, polyarteritis nodosa and ulcerative colitis, all them processes related to climate change, as mentioned above. This disease uses to show a sub-acute visual loss, with central and paracentral scotomas, anterior uveitis, mild vitritis, and large and yellowish-white deep placoid injuries; exceptionally, may result in cerebral vasculitis, and, then, sometimes, in an ictus [57 196 201 246 247 248].<br>**ii) Multiple evanescent white spots syndrome (MEWDS)**. Despite its association to HLA B51 antigen (as occurs in Behçet disease), this is an inflammatory autoimmune disease activated by viral processes and vaccination, showing similar symptoms as Borreliosis. It shows a slight vision reduction, an increase of the blind spot, and an altered visual field with scotomas, but uses to have a good prognosis [57 196 201]. | Environmental factors of several infectious diseases |
| **6. Retina.** | **Related environmental variables** |
| **Tractional Retinal Detachment and Retinal Tears (M).** They are strongly associated with heatwaves (≥25ºC) in patients under 75 years. In addition, other researches point to the increasing of light daily hours; thus, dry years imply, especially in summer, a higher number of Tractional Detachments due to high luminosity. Others relate Retinal Detachment and summer extreme temperatures: differences between maximum and minimum during summer months, low rainfall and high relative humidity (with high perspiration). Lin et al., (2011) [249] have also noted an increase in retinal detachment cases in summer and a decrease from October to February [16 250 251 252]. | Heatwaves<br>Summertime<br>High luminosity<br>Extreme thermal amplitude<br>Low rainfall<br>High relative humidity |

| | |
|---|---|
| **Posterior Vitreous Detachment (N).** It has especially been related to sustained high temperatures [253]. | High temperatures |
| **Age Macular Degeneration (AMD) (O)**. Estelle (2020) [61] related it with UV radiation, and Morris et al. (2007) [254] with sunlight. Others authors [14] highlight a relation AMD-blue light (but not UVA) or a relation AMD-UVB [12]. Tomany et al. (2004) [83] underscore the protecting factor of sunglasses and hats to avoid the early onset of AMD, drusen and retinal pigmentary epithelium disorders. | UVR<br>Sunlight<br>Blue light<br>UVB<br>Sunglasses and hat (protecting factor) |
| **Central serous choroidopathy (P)**. It has been linked to various aetiologies, but lately it has been closely related to *Helicobacter pylori* infection [255] [256], gastric bacteria whose main reservoir is polluted groundwater, which is an increasing element due to climate change [93] [257] [258] by means of torrential rainfall, floods and high temperatures. It is a disease characterized by a serous detachment of macular neuro-sensorial retina, common in white young males and middle-aged women[57] . | Polluted groundwater<br>Torrential rainfall<br>Floods<br>High temperatures |

Note: A, B, C, D… See point 2 of the main text.

**Table S2.** Trends of environmental variables in Southern Spain.

| Environmental Variable | Commentaries |
|---|---|
| Temperatures | It has been identified as a sustained increasing trend of average, minimum and maximum temperatures. Moreover, it has been detected a trend to the higher frequency of heatwaves, number of tropical days and number of torrid days [17 19]. Mean Annual Temperatures 1960-1989: Almería, 18.4°C; Málaga, 17.9°C; 1990-2019: Almería, 18.9°C; Málaga, 18.7°C. |
| Droughts | Greater frequency is expected. A long absence of rainfall tends to be linked to a long absence of clouds (especially vertical clouds, which highly absorb UVR), less humidity, higher temperatures and sun exposure. That increases the intensity of visible, infra-red and ultraviolet radiation, highlighting that an 8.3% of the global radiation which reaches the Earth is UV range and a 25% of this is UVB and UVC, highly harmful for human health [153 154 155]. |
| Forest fires | Higher vulnerability. The higher values of atmospheric $CO_2$ provide a better growing of plants, among them the shrubs, main fuel of forest fires. In addition, higher temperatures increase the evaporation and, thus, the flammability. Forest fires are related to higher $PM_{10}$ concentrations, which are responsible for premature deaths [4 156 157 158]. |
| Floods and erosive processes from pouring rain | Greater frequency is expected. World thresholds in rainfall are different in each region, even within Southern Spain. However, it is common that higher temperatures are joined to more frequent pouring rain, and, because of this, floods frequency is higher. The floods are increasing in Costa del Sol during the last years, and thus, also erosion processes [21 71 72]. |
| Hydric resources | Decreased quantity and quality because of droughts and floods. Due to the above-mentioned floods, it is increasing both damages to infrastructures for drinking water supply and infectious diseases due to waterlogging. This is an actual risk in Southern Spain, where it has been detected several sea intrusions in aquifers [73 159 160 161]. |
| Coastal losses and coastal phenomena | Greater frequency is expected. The rising sea level, higher temperatures and higher winds enhance the frequency of coastal flooding along Malaga shore, adding several related health problems. Moreover, this natural process is compounded by human structures, due to property voracity, especially next to the sea, increasing the number of people which could be affected economically and in health terms [17 74 162 163 164 165 166]. |
| Endangered species and habitats | The trend to be listed as species threatened with extinction category is increasing. Higher frequency of animal displacement to new habitats, with the risk of transmitting new zoonosis. Habitats destruction, pesticides and human pressure enable the proliferation of damaging species as much as they reduce individuals of species in extinction risk, eliminate biodiversity potentially useful for medical research, and trigger zoonosis from the external origin; it is well known that is one of the hypotheses for Covid19 pandemic [167 168 169 170 171]. |
| Water-borne diseases | Higher frequency. Water-borne diseases together other diseases that we thought have been eradicated may reappear because of higher temperatures on standing water, |

| | |
|---|---|
| | allowing microbial proliferation. Even just heavy rain followed by warm temperatures may provide this fact [75, 145, 172]. |
| Others altered variables | Ground-level ozone, pollutants precursors of ground-level ozone, greenhouse gases, acidifying agents, particles below 10 microns ($PM_{10}$) or below 2,5 ($PM_{2.5}$), persistent organic pollutants, benzene, aromatic polycyclic hydrocarbons, dioxins, furans, etc. All of them are toxic elements that use to reach high concentrations in urban areas, especially during stability moments [76, 173]. |
